# Increased yields and biological potency of knob-into-hole-based soluble MHC class II molecules

**DOI:** 10.1038/s41467-019-12902-2

**Published:** 2019-10-29

**Authors:** Pau Serra, Nahir Garabatos, Santiswarup Singha, César Fandos, Josep Garnica, Patricia Solé, Daniel Parras, Jun Yamanouchi, Jesús Blanco, Meritxell Tort, Mireia Ortega, Yang Yang, Kristofor K. Ellestad, Pere Santamaria

**Affiliations:** 1 Institut D’Investigacions Biomèdiques August Pi i Sunyer, Barcelona, 08036 Spain; 2 Julia McFarlane Diabetes Research Centre (JMDRC) and Department of Microbiology, Immunology and Infectious Diseases, Snyder Institute for Chronic Diseases and Hotchkiss Brain Institute, Cumming School of Medicine, University of Calgary, Alberta, T2N 4N1 Canada; 3 Division of Endocrinology, Hospital Clinic i Provincial de Barcelona, Barcelona, Spain; 4 Department of Biochemistry and Molecular Biology, Cumming School of Medicine, University of Calgary, Alberta, T2N 4N1 Canada

**Keywords:** Applied immunology, Autoimmunity, Nanoparticles

## Abstract

Assembly of soluble peptide-major histocompatibility complex class II (pMHCII) monomers into multimeric structures enables the detection of antigen-specific CD4^+^ T cells in biological samples and, in some configurations, their reprogramming in vivo. Unfortunately, current MHCII-αβ chain heterodimerization strategies are typically associated with low production yields and require the use of foreign affinity tags for purification, precluding therapeutic applications in humans. Here, we show that fusion of peptide-tethered or empty MHCII-αβ chains to the IgG1-Fc mutated to form knob-into-hole structures results in the assembly of highly stable pMHCII monomers. This design enables the expression and rapid purification of challenging pMHCII types at high yields without the need for leucine zippers and purification affinity tags. Importantly, this design increases the antigen-receptor signaling potency of multimerized derivatives useful for therapeutic applications and facilitates the detection and amplification of low-avidity T cell specificities in biological samples using flow cytometry.

## Abstract (web summary)

Recombinant MHC class II molecules are instrumental in antigen-specific T-cell identification assays and showed efficacy as experimental medicines. Here, the authors engineer MHC class II molecules with species-specific knob-into-hole heteromerization domains, enabling a translatable purification process with improved stability, yields, and biological potency.

## Introduction

Engineering and expression of soluble MHC molecules displaying specific antigenic epitopes (pMHC) has been instrumental not only for the analysis of antigen-specific CD4^+^ and CD8^+^ T-cell cells within biological samples^1^, but also for the production of pMHC-based compounds capable of modulating immune responses in vivo^2–5^.

Production of soluble pMHC class II molecules is more challenging than production of their pMHC class I counterparts. Assembly of pMHC class I monomers involves the heterodimerization of an MHC class I heavy chain with β2-microglobulin via conserved, non-polymorphic residues in the presence of short (8–10 amino acid-long) peptides capable of binding to a peptide-binding groove located at the amino terminal end of the heavy chain. The rules that govern peptide binding to MHC class I molecules are well defined, including the identification of pockets capable of anchoring specific epitopes via complementary anchor residues that fix their binding register and stabilize the trimolecular complex. These structural hallmarks facilitate the expression and purification of stable pMHC class I monomers from eukaryotic expression systems, as well as the re-folding and assembly of the trimolecular complex in vitro, using peptide and heavy and light chains expressed separately in prokaryotic expression systems.

Expression of MHC class II molecules has been significantly more difficult because secreted MHC class II α and β chains lacking the transmembrane and cytoplasmic domains do not form stable heterodimers, even in the presence of high affinity peptide ligands. This is because the two α-helical transmembrane domains of the MHC class II α and β chains play key roles in the proper assembly and expression of stable heterodimers on the cell surface^6^. This challenge was addressed by replacing the transmembrane and cytoplasmic domains of MHC class II chains by leucine zipper motifs (reviewed elsewhere^7^). However, since MHC class II binding peptides play a critical role in the assembly and stabilization of the αβ heterodimer, these approaches do not invariably support the expression of pMHC monomers displaying epitopes with low affinity for MHC and/or the expression of MHC class II types with peculiar structural features, such as certain HLA-DQ molecules. This represents a fundamental limitation for the use of these reagents as a tool to enumerate and track cognate autoreactive T-cells in autoimmunity, where many naturally occurring autoimmune disease-relevant epitopes are weak MHC binders. This limitation is compounded by the fact that MHC class II (as opposed to class I) molecules can bind peptides of various lengths and on various registers through alternate anchor residues.

Another significant limitation of current soluble pMHC class II engineering approaches is that they are not currently suited for the production of multimeric pMHC class II-based compounds at scale for therapeutic purposes^2,4,5^. This is so because there are no orthogonal chromatographic separation schemes capable of purifying pMHC class II complexes from eukaryotic cell culture supernatants with the degree of purity, cost and yields required for clinical translation. Although for pure experimental purposes, this caveat can be addressed by addition of affinity separation tags into the pMHC complex, this practice is not acceptable for human translation as it bears the unacceptable risk of triggering the generation of anti-drug antibodies.

Here, we set out to engineer a generalizable molecular design capable of addressing these limitations. We report a new molecular pMHC class II heterodimerization design based on the “knob-into-hole” (KIH) approach used for the generation of bi-specific monoclonal antibodies^8,9^. This approach allows stable alpha/beta heterodimerization for a broad range of MHC class II subtypes, with increased molecular stability, production yields and antigen-receptor binding and triggering potency. These compounds are also amenable to various modalities of signal amplification of multimer-based enumeration of cognate T-cells, and to the production of “empty” heterodimers suitable for peptide library screens.

## Results

### A mammalian expression system for soluble pMHC class II

**Fig. 1:**
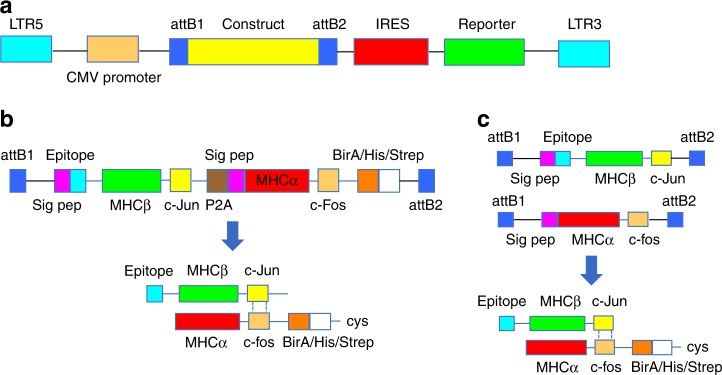
pMHCII-expression construct design. Cartoons depict the general structure of the lentiviral system (**a**) and the type of constructs used (**b**, **c**). **b** Structure of a P2A-linked pMHCβ and MHCα chain-coding construct (top) and a representation of the resulting pMHCII product secreted into the cell culture supernatant (bottom). **c** Single pMHCβ- or MHCα chain constructs that were serially transduced into CHO cells to produce the resulting pMHCII αβ heterodimers (bottom)

**Fig. 2:**
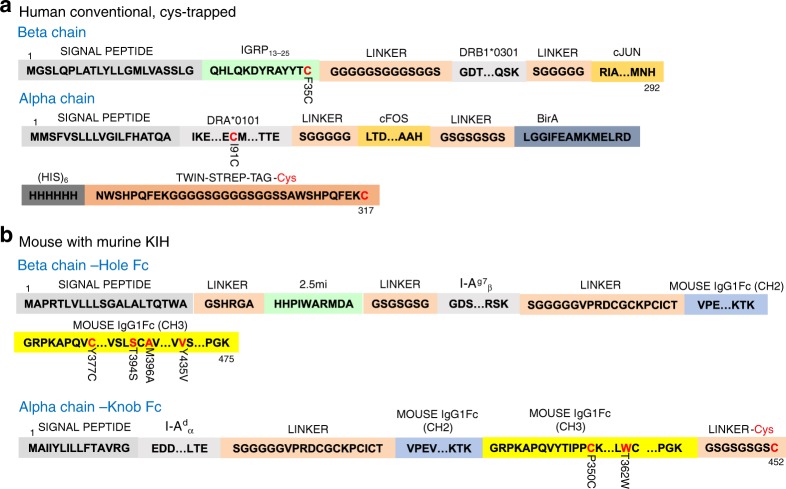
Key junctional, linker and motif sequences for cys-trapped human pMHCII and mKIH-based mouse pMHCII. **a** Key amino acid sequences of human pMHCII molecules encoding a cys-trapped IGRP_13–25_/DRA1*0101/DRB1*0301 pMHCII heterodimerized via c-jun/c-fos leucine zippers (also referred to “conventional”). ‘…’ is used to indicate that corresponding intervening amino acid sequences are not shown, as they are publicly available. Residues in red are mutated and the original residue and its position are indicated immediately below. **b** Key amino acid sequences of a mouse pMHCII molecule encoding BDC2.5mi/IAα^d^/IAβ^g7^ heterodimerized using a carboxyterminal mouse IgG1-Fc-based KIH

**Fig. 3:**
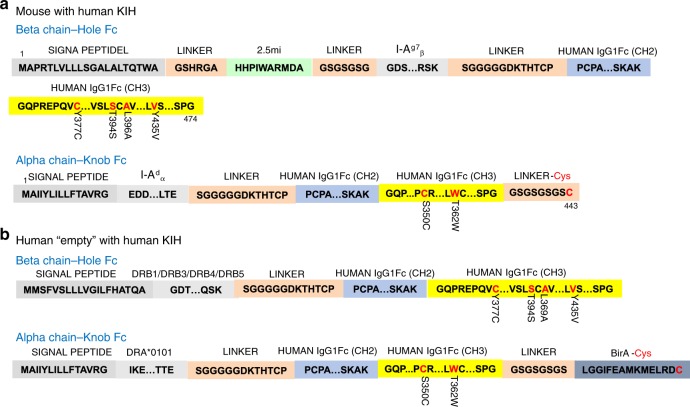
Key junctional, linker and motif sequences for hKIH-based mouse pMHCII and empty hKIH-based human pMHCII. **a** Key amino acid sequences of a mouse pMHCII molecule encoding BDC2.5mi/IAα^d^/IAβ^g7^ heterodimerized using a carboxyterminal human IgG1-Fc-based KIH. **b** Key amino acid sequences for “empty” human MHCII molecules encoding DRA*0101 MHCα and DRB1, DRB3, DRB4 or DRB5 MHCβ chains heterodimerized using a carboxyterminal human IgG1-Fc-based KIH

**Fig. 4:**
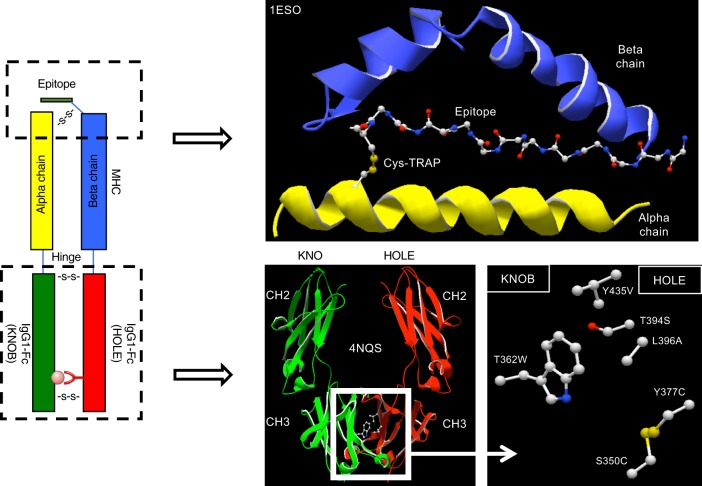
Cartoons depicting the structure of the KIH-based pMHCII constructs or specific domains. Left: primary structure of a Cys-trapped KIH-based pMHCII heterodimer. Top right: secondary structure of the peptide-binding domain loaded with a peptide bound to the MHCII molecule on a specific register via a disulfide bridge between the carboxyterminal end of the peptide and a complementary Cys on the MHCII α chain. Bottom right: predicted quaternary structure of the KIH Fc portion of the KIH-based constructs and the key amino acid substitutions that were used to promote KIH-based heterodimerization

We used lentiviral vectors encoding IRES-CFP or IRES-EGFP reporter cassettes (Fig. 1a) to express pMHCIIs in CHO cells. The pMHCII α and β chains were either transcribed from a single ORF as two chains separated by a P2A ribosomal skipping sequence (Fig. 1b), or from two different ORFs in different vectors (Fig. 1c). Figures 2–4 summarize the structural features of representative constructs for the various pMHCIIs described here, as well as key junctional sequences. Table 1 provides a list of the mouse and human pMHCIIs used here and their expression yields. Briefly, transduced CHO-S cells expressing high levels of EGFP and CFP were sorted by flow cytometry and grown in protein-free media in shake flasks using a fed batch protocol. pMHCIIs were purified from supernatants and used directly to coat iron oxide nanoparticles (NPs), or were biotinylated to produce pMHCII tetramers.

**Table 1:** Peptides, MHC molecules, heterodimerization domains and yields

Tethered epitope	Sequence	MHC beta	MHC alpha	Heterodimers	Yield (mg/L)
BDC.2.5mi	HHPIWARMDA	I-A_β_^g7^	I-A_α_^d^	JUN/FOS	17.7
BDC.2.5mi	HHPIWARMDA	I-A_β_^g7^	I-A_α_^d^	HOLE/KNOB	80.6
TOPO(722–736)	KLNYLDPRITVAWCK	I-A_β_^b^	I-A_α_^b^	JUN/FOS	2.3
ApoB(3501–3516)	SQEYSGSVANEANVY	I-A_β_^b^	I-A_α_^b^	JUN/FOS	0.38
mDSG3(301–315)	RNKAEFHQSVISQYR	I-A_β_^b^	I-A_α_^b^	JUN/FOS	0.2
IGRP(13–25)	QHLQKDYRAYYTF	DRB1*0301	DRA*0101	JUN/FOS	12
IGRP(13–25) cys-trap	QHLQKDYRAYYTC	DRB1*0301	DRA*0101	JUN/FOS	4
IGRP(13–25)	QHLQKDYRAYYTF	DRB1*0301	DRA*0101	HOLE/KNOB	32.8
IGRP(13–25) cys-trap	QHLQKDYRAYYTC	DRB1*0301	DRA*0101	HOLE/KNOB	95.8
PPI(76–90)88S cys-trap	SLQPLALEGSLQSRC	DRB1*0401	DRA*0101	JUN/FOS	4.12
PPI(76-90)88S	SLQPLALEGSLQSRG	DRB1*0401	DRA*0101	HOLE/KNOB	44.5
IGRP(23–35)	YTFLNFMSNVGDP	DRB1*0401	DRA*0101	JUN/FOS	0.6
IGRP(23–35) cys-trap	YTFLNFMSNVGDC	DRB1*0401	DRA*0101	JUN/FOS	45.9
Glia(62–72) cys-trap	PQPELPYPQPC	DQB1*0201	DQA1*0501	JUN/FOS	2.6
Glia(62–72)	PQPELPYPQPE	DQB1*0201	DQA1*0501	HOLE/KNOB	30.4
Glia(62–72) cys-trap	PQPELPYPQPC	DQB1*0201	DQA1*0501	HOLE/KNOB	39.5
NONE	–	DRB1*0301	DRA*0101	HOLE/KNOB	10.2
NONE	–	DRB4*0101	DRA*0101	HOLE/KNOB	29.3
NONE	–	DRB5*0101	DRA*0101	HOLE/KNOB	29.4
NONE	–	DRB1*1501	DRA*0101	HOLE/KNOB	22.9

This Table was generated using contemporary CHO cell cultures using representative cell lines

### Leucine zipper-based pMHCII have variable stability

**Fig. 5:**
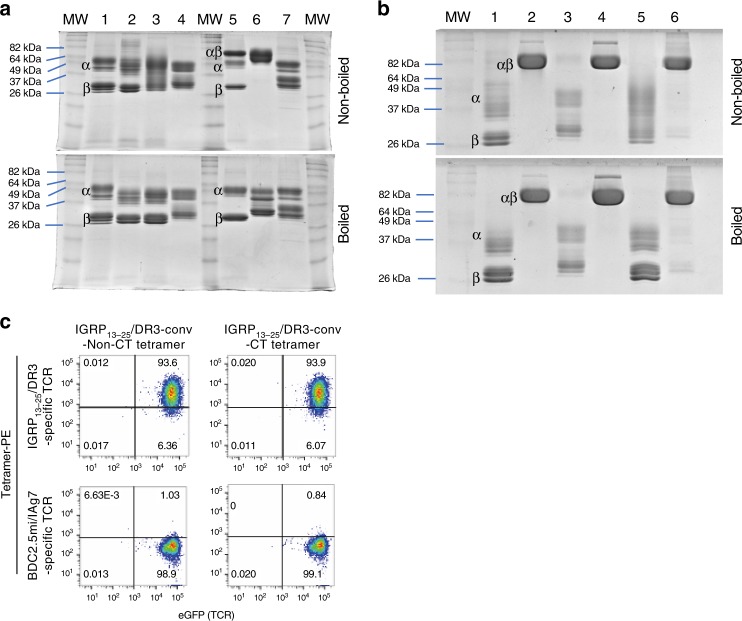
Stabilization of pMHCII heterodimers by introduction of peptide-MHCα chain Cys-traps. **a** SDS-PAGE for various c-jun/c-fos-based pMHCII heterodimers carrying or lacking Cys-traps (CT), under native vs. denaturing conditions. 1: BDC2.5mi/IA^g7^; 2: IGRP_13–25_/DR3; 3: PPI_(76–90)(88S)_/DR4; 4: IGRP_23–35_/DR4; 5: Topo_722–736_/IAb; 6: ApoB_3501–3516_/IA^b^; 7: DSG3_301–315/_IA^b^. MW, molecular weight markers. Except for ApoB_3501–3516_/IA^b^, all other pMHCII heterodimers shown are partially or completely SDS unstable. The electrophoretic behaviors of the various pMHCs shown herein were consistent with those seen with earlier preparations of the same proteins that had been previously run in separate gels at least once before. They were re-expressed, re-purified and re-run again together to generate this figure. **b** Effects of Cys-trapping on SDS stability of pMHCII heterodimers. Data correspond to 1: IGRP_13–25_/DR3-non-CT; 2: IGRP_13–25_/DR3-CT; 3: IGRP_23–35_/DR4-non-CT; 4: IGRP_23–35_/DR4-CT; 5: PPI_76–90(88S)_/DR4-non-CT; and 6: Glia_62–72_/DQ2-CT. **c** pMHCII tetramer/CD4 FACS dot plots for Jurkat cells expressing human CD4 and an IGRP_13–25_/DR3-specific TCR (top) or mouse CD4 and a BDC2.5mi/IA^g7^-specific TCR (bottom) stained with non-CT (left) or CT (right) IGRP_13–25_/DR3 tetramers. The staining patterns shown are representative of at least two independent experiments

It has been shown that the epitope is a major stabilizer of soluble pMHCII heterodimers^10,11^. Peptides binding with high affinity support higher heterodimer stability than those binding with low affinity. However, intrinsic molecular properties of allelic MHCII molecules also play a major role in defining the stability of pMHCIIs, independently of the peptide^12,13^. As a result, whereas certain pMHCII molecules migrate as a single, large molecular species in non-denaturing SDS-PAGE, most others melt into single α and β chains (Fig. 5a) and are expressed at low yields (Table 1).

### Cys-trapped pMHCII heterodimers increase pMHC stability

We reasoned that we could increase the stability and possibly the production yields of SDS-unstable c-jun/c-fos-zippered MHCIIs (herein also referred to ‘conventional’) by introducing cysteines at appropriate positions in the peptide and the MHCII α chain to anchor the peptide onto the MHC on a preferred binding register^14,15^ (herein referred to as cys-trapping (CT)). We note that our prior attempts to address this issue by introducing artificial disulfide bonds at or near the c-jun/c-fos zipper in poorly expressing pMHCII constructs were unsuccessful. We first focused on the type 1 diabetes (T1D)-relevant IGRP_13–25_/DRB1*0301/DRA1*0101 complex (Table 1). We replaced a C-terminal phenylalanine in IGRP_13–25_ and a proximal serine in the MHCII α chain for cysteines (Figs. 2–4). This resulted in SDS stability (Fig. 5b) without any appreciable loss of cognate T-cell binding efficiency, as measured using pMHC tetramers and a human CD4/TCR-transduced Jurkat cell line (Fig. 5c). Similar results were obtained with other pHLA molecules, such as IGRP_23–35_/DRB1*0401/DRA1*0101 (Fig. 5b). The use of a cys-trap also enabled the production of much more difficult-to-express HLA molecules, such as HLA-DQB1*0201/DQA1*0501 displaying gliadin residues 62–72 (Table 1 and Fig. 5b). Cys-trapping, however, increased production yields for some but not all pMHCs (e.g., IGRP_13–25_/DRB1*0301/DRA1*0101) (Table 1). Furthermore, cys-trapping cannot be adopted by all pMHCIIs, because introduction of artificial cysteines within the peptide might in some cases impair T-cell binding and/or activation, and because epitopes that already contain naturally occurring cysteines within their sequence are not suitable for this approach.

### A knob-into-hole-based pMHCII design

To address this and other limitations of current pMHCII production strategies, including heterodimer instability, discrete production yields, and the lack of efficient and scalable purification schemes broadly applicable to any pMHC type (for human in vivo use), we explored the feasibility of using a knob-into-hole (KIH)-IgG-based heterodimerization strategy. Introduction of complementary amino acid substitutions in the CH3 domain of the Fc region of human IgG1 (or other IgG subtypes) results in the generation of two different Fc molecules (knob and hole) with favorable heterodimerization and unfavorable homodimerization potential^8,9^. We reasoned that, unlike Fc-fusion-based pMHC dimerization, which generates large Ig-like molecular structures in which αβ heterodimer formation and stability still require the use of leucine zippers and are regulated by the same principles that control the assembly of non-Fc-fused, c-jun/c-fos zippered pMHCIIs^16–19^, KIH-based pMHCII heterodimerization would potentially render pMHCIIs intrinsically more stable with only a relatively minor increase in total molecular weight.

**Fig. 6:**
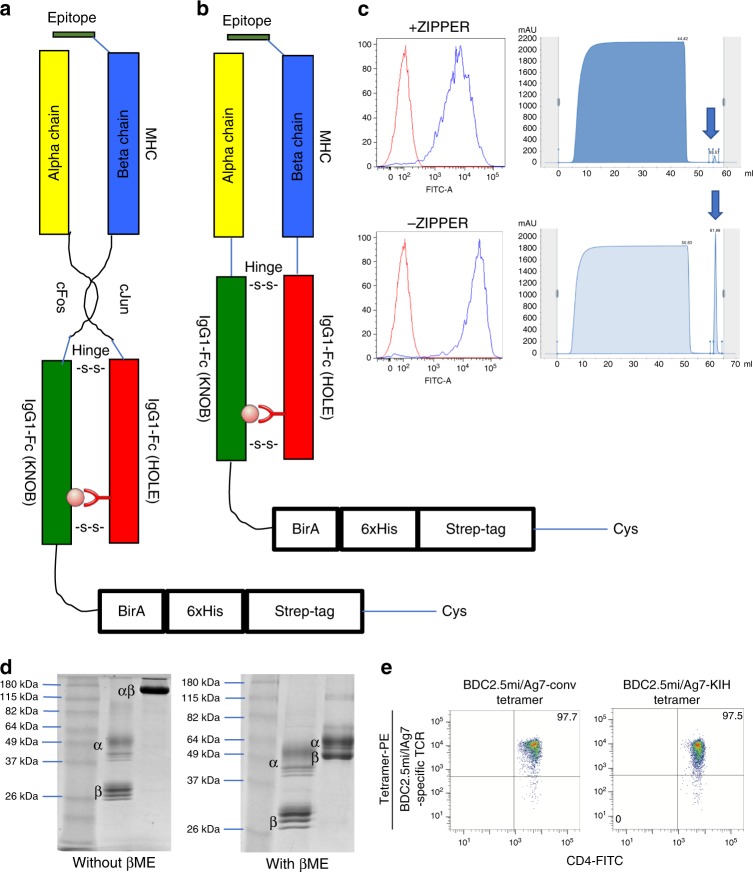
Use of a c-jun/c-fos zipper in KIH-based pMHCII is incompatible with expression. **a**, **b** Cartoons displaying the structure of the two types of KIH constructs tested here. **c** (left), Expression of eGFP in CHO-S cell lines transduced with lentiviruses encoding the constructs depicted in A (top) and B (bottom), indicating adequate construct transcription and translation. **c** (right), FPLC elution profiles of pMHC class II from Strep-tactin columns loaded with supernatants from CHO cells expressing the constructs in A (top) or B (bottom). **d** Effects of the KIH on the SDS stability of a representative pMHCII heterodimer (out of at least 10 different KIH-based pMHCs), in the absence of Cys-trapping. Data correspond to c-jun/c-fos-based BDC2.5mi/IA^g7^ (‘conv’, left lane) and a KIH-based BDC2.5mi/IA^g7^ (right lane). βME, beta-mercaptoethanol. **e** Representative pMHCII tetramer/eGFP (TCR) FACS dot plots for BDC2.5-TCR-transgenic CD4^+^ T-cells stained with c-jun/c-fos- (‘conv’, left) or KIH-based BDC2.5mi/IA^g7^ tetramers (right). The staining patterns shown are representative of at least two independent experiments

We tethered the mouse IAα^d^ chain with a modified Fc region of human IgG1 to behave as a knob (both with and without the c-fos motif), and the corresponding IAβ^g7^ chain (with and without the c-jun motif) to the Fc region of human IgG1 modified to behave as a hole (Figs. 3a and 6a, b). In our initial designs, we also included a BirA biotinylation site, a 6× histidine and twin strep tags, and a cysteine at the C-terminal end of the knob, generating a ‘knob’ that is larger than its ‘hole’ counterpart. Both the leucine-zippered and non-zippered cell lines expressed the transgenic RNA, as documented by the expression of EGFP (Fig. 6c, left), but only the latter secreted protein G-binding material in the supernatant (Fig. 6c, right), which ran as a single band in native SDS-PAGE (Fig. 6d, left panel), and as two separate bands of different molecular weight, as expected, but similar intensity in denaturing SDS-PAGE (Fig. 6d, right panel), suggesting ~1:1 stoichiometries. The KIH version of this pMHCII expressed at >4-fold higher levels than its non-KIH-based counterpart (Table 1). These molecules folded appropriately because pMHCII tetramers generated with these KIH-based pMHCII monomers stained splenic CD4^+^ T-cells from a transgenic mouse expressing a BDC2.5mi-specific T-cell receptor (TCR) essentially like its zippered, non-KIH-based counterpart (Fig. 6e).

### Receptor-signaling properties of hIgG1 KIH-based pMHCII

When delivered systemically, NPs coated with autoimmune disease-relevant pMHCII (pMHC-NP) can reprogramme (and expand) autoantigen-experienced effector/memory T-cells into cognate T-regulatory type 1 (TR1) cells, leading to reversal of various autoimmune diseases^2,5^. The biological potency of these compounds (TR1 cell formation in vivo) is a function of pMHC valency on the NP surface and can be gauged in vitro using reporter cell lines^4^.

**Fig. 7:**
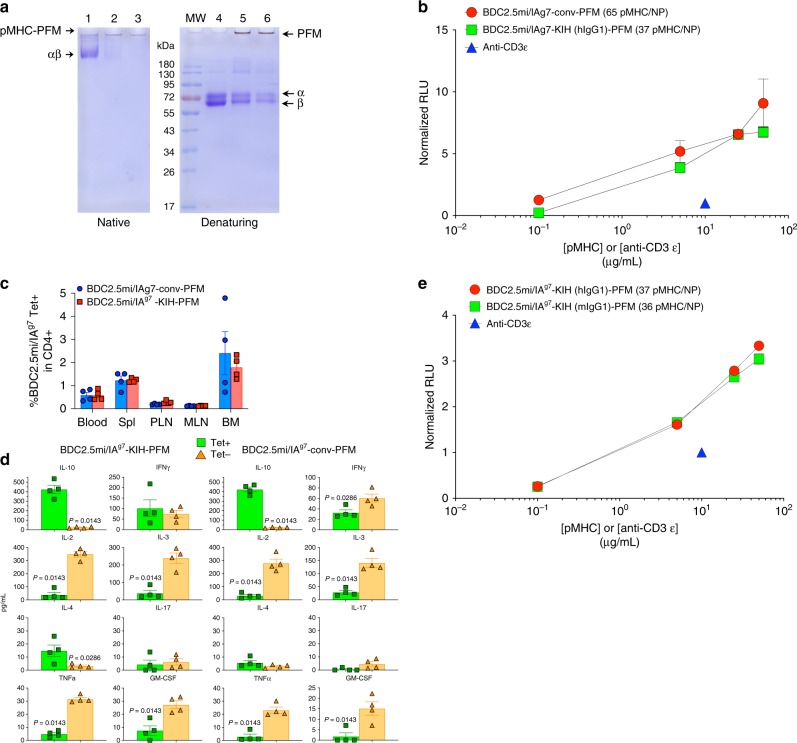
Biological potency of NPs displaying KIH- vs. c-jun/c-fos-based pMHCII. **a** Native (left) and denaturing (right) SDS-PAGE for NPs coated with a representative KIH-based pMHCII molecule. PFM denotes the iron oxide NP. MW: molecular weight markers; 1: 2 μg of KIH-based BDC2.5mi/IA^g7^ monomers; 2: 2.2 μL of PFM coated with KIH-based BDC2.5mi/IA^g7^ monomers; 3: 1.1 μL of PFM coated with KIH-based BDC2.5mi/IA^g7^ monomers; 4: 2 μg of KIH-based BDC2.5mi/IA^g7^ monomers; 5: 2.2 μL of PFM coated with KIH-based BDC2.5mi/IA^g7^ monomers; 6: 1.1 μL of PFM coated with KIH-based BDC2.5mi/IA^g7^ monomers. Electrophoretic behavior of the KIH-based pMHCII-NP compound shown herein is representative of at least 10 different pMHCII-NP preparations made using KIH-based pMHCIIs. **b** Luciferase activity induced by NPs coated with c-jun/c-fos- (‘conv’) or KIH-based BDC2.5mi/IA^g7^ monomers (normalized to that induced by soluble anti-CD3ε mAb) on Jurkat cells co-expressing mouse CD4, a BDC2.5 mi/IA^g7^-specific TCR and an NFAT-driven luciferase reporter. Data correspond to mean ± SEM of triplicates. **c** Percentages of BDC2.5mi/IA^g7^ tetramer-positive CD4^+^ T-cells in blood, spleen, pancreatic lymph nodes (PLN), mesenteric lymph nodes (MLN) and bone marrow (BM) from NOD mice treated (twice a week for 5 weeks) with NPs coated with c-jun/c-fos-based (‘conv’) BDC2.5mi/IA^g7^ or KIH-based BDC2.5mi/IA^g7^ monomers (20 μg pMHC/dose). Data correspond to average ± SEM values from 4 mice/group. **d** Cytokine profile of the tetramer^+^ cells isolated from the mice in (**c**). Tetramer^+^ cells were challenged with anti-CD3/anti-CD28 mAb-coated beads for 3 days and the supernatants assayed for cytokine content using Luminex technology. Data correspond to average ± SEM values of cells isolated from 4 mice/group. *P* values were calculated via Mann–Whitney U and are considered significant if *P* < 0.05. **e** Luciferase activity induced by NPs coated with KIH-based BDC2.5 mi/IA^g7^ pMHCs carrying a mouse or a human Fc-based KIH (normalized to that induced by soluble anti-CD3ε mAb) on Jurkat cells co-expressing mouse CD4, a BDC2.5mi/IA^g7^-specific TCR and an NFAT-driven luciferase reporter. Data correspond to mean ± SEM of triplicates. Source data for panels (**b**–**e**) are provided as a Source Data File

We coupled these molecules to maleimide-functionalized NPs via their C-terminal free cysteine as described in ref. ^4^. As shown in Fig. 7a, most of the pMHC in the preparations remained coupled to the NPs when electrophoresed in SDS-PAGE under native conditions, but were released (as NP-free PEG-pMHC conjugates) under denaturing conditions. Quantification of the pMHC valencies of these compounds indicated that, in general, these ~50% larger KIH-based pMHC monomeric structures coat at lower valencies (~20% fewer) than their conventional non-KIH-based counterparts (45 ± 2.8 vs. 36 ± 2.5; *n* = 4 and 5, respectively).

**Fig. 8:**
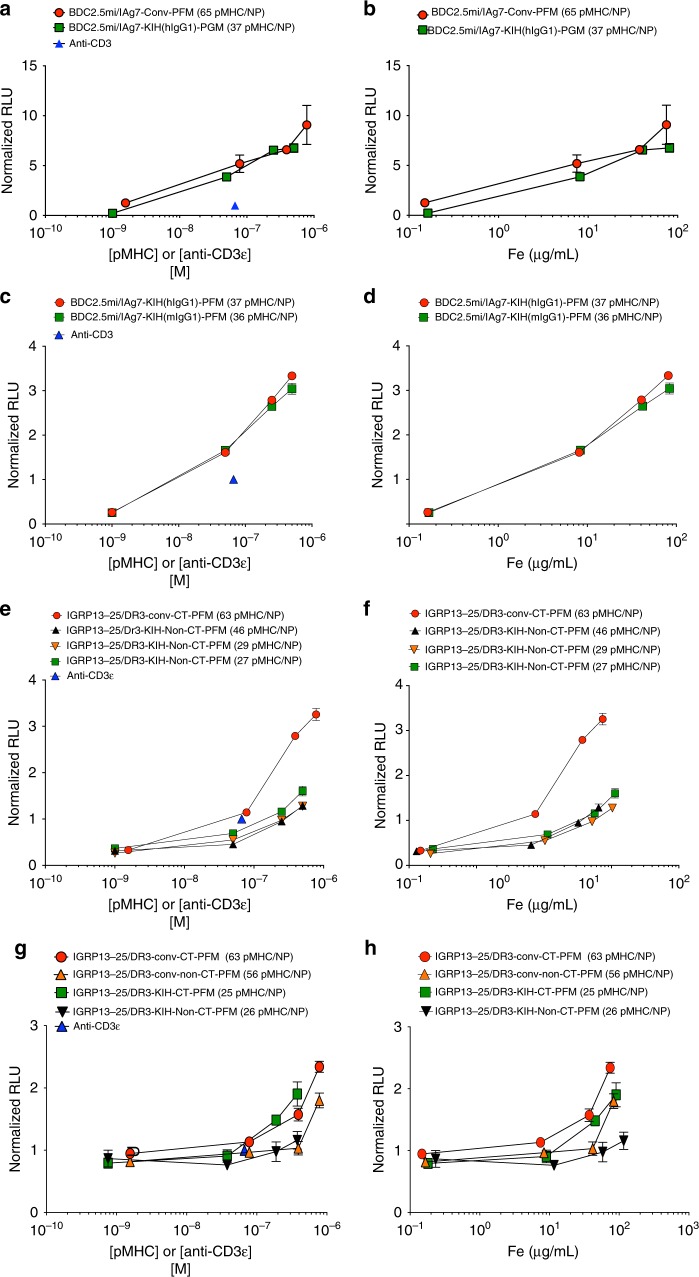
TCR signaling potency of pMHC-NPs as a function of pMHCII molarity or NP number. **a**, **b** Luciferase activity induced by NPs coated with c-jun/c-fos- (‘conv’) or KIH-based BDC2.5mi/IA^g7^ monomers (normalized to that induced by soluble anti-CD3ε mAb) on Jurkat cells co-expressing mouse CD4, a BDC2.5mi/IA^g7^-specific TCR and an NFAT-driven luciferase reporter. Data correspond to main Fig. 7b but normalized by molar concentration of pMHCII or NP number. **c**, **d** Luciferase activity induced by NPs coated with KIH-based BDC2.5mi/IA^g7^ pMHCIIs carrying a mouse or a human Fc-based KIH (normalized to that induced by soluble anti-CD3ε mAb) on Jurkat cells co-expressing mouse CD4, a BDC2.5mi/IA^g7^-specific TCR and an NFAT-driven luciferase reporter. Data correspond to main Fig. 7e but normalized by molar concentration of pMHCII or NP number. **e**, **f** Luciferase activity induced by NPs coated with c-jun/c-fos-based (‘conv’), Cys-trapped IGRP_13–25_/DR3 pMHCs vs. NPs coated with non-Cys-trapped KIH-based IGRP_13–25_/DR3 coated at three different valencies on Jurkat cells co-expressing human CD4, an IGRP_13–25_/DR3-specific TCR and an NFAT-driven luciferase reporter. Data correspond to main Fig. 9d but normalized by molar concentration of pMHCII or NP number. **g**, **h** Luciferase activity induced by NPs coated with c-jun/c-fos-based/Cys-trapped or KIH-based/Cys-trapped IGRP_13–25_/DR3 monomers vs. their non-Cys-trapped counterparts on Jurkat cells co-expressing human CD4, an IGRP_13–25_/DR3-specific TCR and an NFAT-driven luciferase reporter. Data correspond to main Fig. 9e but normalized by molar concentration of pMHCII or NP number. Data correspond to mean ± SEM of triplicates. Source data are provided as a Source Data File

We next compared the TCR signaling potency of NPs coated with non-KIH-based BDC2.5mi/IA^g7^ pMHC (at 65 pMHCs/NP) with NPs coated with its KIH-based counterpart (at 37 pMHCs/NP) (Fig. 7a), on Jurkat cells co-expressing mouse CD4, a cognate TCR and NFAT-driven luciferase. Both compounds had similar potency, despite carrying significantly different pMHC valencies (Figs. 7b and 8a, b). This suggested that these KIH-based pMHC-NP structures might perform optimally at pMHC valencies falling below the minimal optimal pMHC valencies defined for the conventional pMHC design^4^.

### In vivo biological activity of hIgG1 KIH-based pMHCII-NPs

Having shown that pMHC-NPs produced using KIH-based pMHCs have adequate TCR-binding and signaling potency, we sought to confirm that these compounds could also trigger the formation and expansion of cognate TR1 cells in vivo, as is the case for compounds produced using conventional pMHCs. As shown in Fig. 7c, the KIH-based pMHCII-NP compounds triggered the formation and expansion of similar numbers of cognate (tetramer^+^) TR1 cells as their non-KIH-based counterparts. Furthermore, the cognate (tetramer^+^) CD4+ T-cells expanding in vivo in response to both types of compounds secreted TR1-relevant cytokines upon stimulation with anti-CD3 and anti-CD28 mAbs ex vivo, as compared with their tetramer^−^ CD4^+^ T-cells (Fig. 7d). Thus, these zipper-less KIH-based pMHCs heterodimers have similar biological activity than the zippered conventional pMHC.

### Biological in vitro properties of mIgG1 KIH-based pMHCII

The heterodimerization potential of a mouse IgG1-based KIH structure has not been previously described. We thus explored the possibility of generating BDC2.5-I-Aβ^g7^-Hole/I-Aα^d^-Knob heterodimers in which the knob-Fc and hole-Fc are derived from the mIgG1-Fc sequence (mKnob and mHole, respectively). This mouse KIH approach would help reduce the unwanted immunogenicity of these compounds used for in vivo experimentation in mice. To this end, we identified the mouse residues that could generate functional mKnob and mHole molecules upon modification (Fig. 2b). The banding patterns of the protein G-purified molecules in denaturing SDS-PAGE gels was essentially identical to those seen for their hIgG1 KIH-based counterparts. In addition, NPs coated with these molecules had quantitatively similar antigen-receptor-signaling properties as NPs coated with their hIgG1 KIH-based counterparts (Figs. 7e and 8c, d).

### Production of hIgG1 KIH-based human pMHCII

**Fig. 9:**
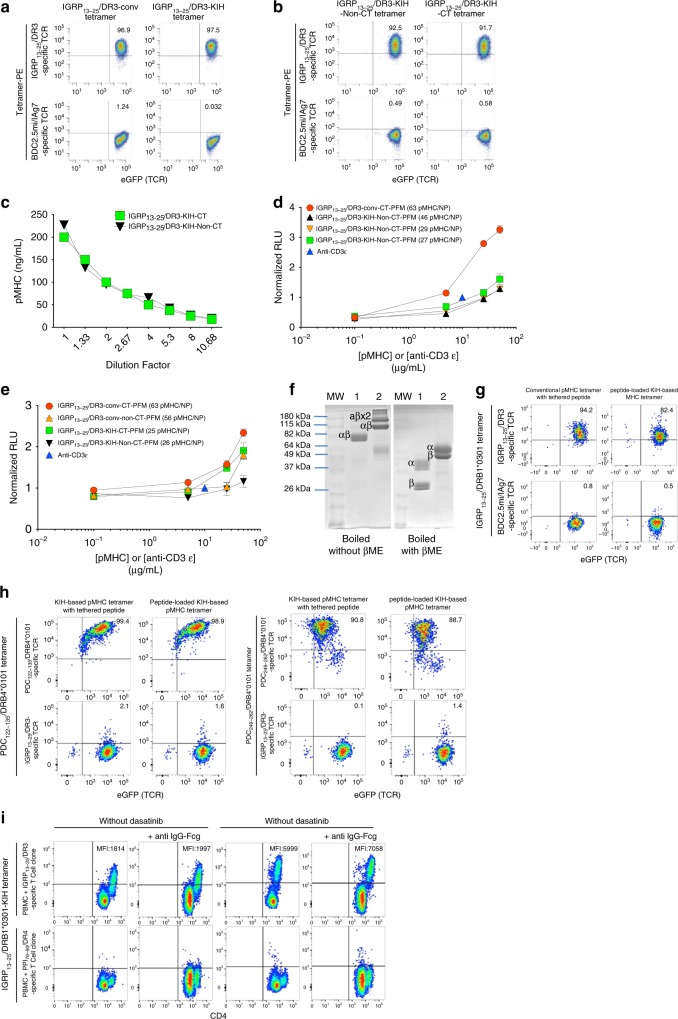
The KIH Fc affords increased biological potency and stabilizes “empty” MHCII. **a** Representative pMHCII tetramer/eGFP (TCR) FACS dot plots for Jurkat cells expressing hCD4 and an IGRP_13–25_/DR3-specific TCR or mCD4 and a BDC2.5mi/IA^g7^-specific TCR (negative control). **b** Representative pMHCII tetramer/eGFP (TCR) dot plots for the Jurkat cells in A, but stained with KIH-based tetramers lacking (left) or carrying a CT (right). **c** Introduction of a CT into KIH-based human pMHCII does not alter their reactivity with a MHCII conformational epitope-specific mAb, as measured by ELISA. Data correspond to mean ± SEM of triplicates. **d** Luciferase activity induced by NPs coated with c-jun/c-fos-based (‘conv’), CT IGRP_13–25_/DR3 pMHCs vs. NPs coated with non-CT, KIH-based IGRP_13–25_/DR3 coated at three different valencies on Jurkat cells co-expressing hCD4, an IGRP_13–25_/DR3-specific TCR and NFAT-luciferase. Data correspond to mean ± SEM of triplicates. **e** Luciferase activity induced by NPs coated with c-jun/c-fos-based/CT (‘conv’) or KIH-based/CT IGRP_13–25_/DR3 monomers vs. their non-CT counterparts on Jurkat cells co-expressing hCD4, an IGRP_13–25_/DR3-specific TCR and NFAT-luciferase. Data correspond to mean ± SEM of triplicates. **f** SDS-PAGE of CT leucine-zippered (1) or KIH-based (2) Gliadin_62–72_/DQB1*0201/DQA1*0501 monomers. βME, beta-mercaptoethanol. **g** Representative pMHCII tetramer/CD4 dot plots for Jurkat cells expressing hCD4 and an IGRP_13–25_/DR3-specific (top) or mCD4 and a BDC2.5mi/IA^g7^-specific TCR (bottom) stained with c-jun/c-fos-based (‘conv’) IGRP_13–25_/DR3 tetramer (peptide-linked; left) or tetramers generated using peptide-loaded empty DR3-KIH monomers (right). **h** Representative pMHCII tetramer/eGFP (TCR) plots for Jurkat cells expressing hCD4 and PDC-E2_122–135_/DRB4-specific TCR (top left), a PDC-E2_249–262_/DRB4-specific TCR (top right) or an IGRP_13–25_/DR3-specific TCR (bottom; negative control). **i** Signal amplification of KIH-based tetramer binding using anti-hFc. Human PBMCs (10^6^) were spiked with cells from a hIGRP_13–25_/DR3-specific T-cell clone (top) or an irrelevant (PPI_(76–90(88S)_/DR4-specific) clone (10^4^) (bottom). Cells were treated with Dasatinib (right) or left untreated (left) and then stained with PE-labeled tetramers and PE-labeled anti-IgG. Values on the plots correspond to the geometric mean fluorescence intensity for pMHC tetramer staining. The staining patterns shown in (**a**), (**b**), (**g**), and (**h**) are representative of at least two independent experiments. Source data for panels (**c**–**e**) are provided as a Source Data File

We next asked if this KIH strategy could also be used to stabilize weaker peptide:MHC interactions, such as IGRP_13–25_/DRB1*0301/DRA1*0101. As was the case for zippered BDC2.5-I-Aβ^g7^-Hole/I-Aα^d^-Knob heterodimers, zippered IGRP_13–25_-DRB1*0301-Hole/DRA1*0101-Knob heterodimers could not be expressed, but removal of the c-jun/c-fos zipper from the molecule led to efficient expression, at levels significantly greater than those obtained from CHO-S cells secreting non-KIH-based IGRP_13–25_-DRB1*0301/DRA1*0101 heterodimers (Table 1). pMHCII tetramers produced with the KIH-based monomers stained cognate T-cells essentially like tetramers produced using leucine-zippered pMHCII monomers (Fig. 9a). Addition of the cys-trap register fixing mutations in the peptide and MHCII α chain of these complexes (Figs. 2a and 4) further increased expression yields (Table 1). This molecular modification did not disrupt the TCR-binding properties of these molecules because staining of cognate TCR-expressing Jurkat cells with tetramers made with the CT vs non-CT KIH-based constructs was essentially equivalent (Fig. 9b). Furthermore, these molecules reacted quantitatively equally to an anti-DR mAb (clone L243) that binds to a conformational epitope on the HLA-DRα chain that requires the correct folding of the αβ heterodimer^20,21^ (Fig. 9c).

### Cys-trapping augments the potency of KIH-based pMHCII

The above data suggested that introduction of a cys-trap between IGRP_13–25_ and DR3 increases the structural stability of the heterodimer and pMHC production yields without interfering with TCR binding. However, when we compared the in vitro potency of NPs coated with a cys-trapped version of the non-KIH-based human IGRP_13–25_/DRB1*0301-DRA*0101 pMHC (at 63 pMHCs/NP) with that of NPs coated with three non-cys-trapped KIH-based IGRP_13–25_/DRB1*0301-DRA*0101 preparations (at 46, 29, and 27 pMHCs/NP), the latter three elicited significantly reduced luciferase responses from cognate Jurkat cells (Figs. 8e, f and 9d). The following three lines of evidence suggested the possibility that these differences might be accounted for by the presence of the cys-trap in the non-KIH-based pMHC that was used as a control. First, NP preparations displaying low valencies of the KIH-based BDC2.5mi/IA^g7^ pMHC performed essentially like NPs displaying high valencies of its zippered, non-KIH-based counterpart (Figs. 2b and 8a, b), suggesting that KIH-based pMHCs support increased TCR signaling. Second, all three NP preparations displaying the non-cys-trapped KIH-based IGRP_13–25_/DRB1*0301-DRA*0101 also performed similarly in this assay, in a valency-independent manner (from 27–46 pMHCs/NP), consistent with the hypothetical increased potency of KIH-based designs.

To investigate this hypothesis, we compared the biological potency of NPs coated with cys-trapped and non-cys-trapped versions of both types of pMHC constructs (non-KIH-based, and KIH-based). Surprisingly, with both construct types, inclusion of a cys-trap boosted potency (Figs. 8g, h and 9e). Indeed, NPs coated with the cys-trapped KIH-based construct had similar function as NPs coated with the cys-trapped non-KIH-based construct, despite significant differences in pMHC valency (56 and 63 for cys-trapped and non-cys-trapped non-KIH-based pMHC, respectively, vs. 25 and 26 for cys-trapped and non-cys-trapped KIH-based pMHC, respectively), again supporting the idea that the use of KIH-based pMHCs on NPs lowers the pMHC valency threshold required for biological activity.

Together, these unexpected observations suggest that the peptide-binding cleft in some non-cys-trapped pMHC monomers (whether leucine zipper (conventional) or KIH-based) might be occupied by endogenous CHO-S peptides, as opposed to the tethered peptides encoded in the expression constructs. Alternatively, this approach generates a more compact pMHC structure that somehow enhances cognate TCR engagement. The increased biological potency of the KIH-based design, particularly when combined with peptide cys-trapping, suggest the possibility that the KIH halve of these pMHC class II molecules may have an allosteric effect on the pMHC interface in a way that improves TCR engagement. Alternatively, this effect might be due to the particular topology adopted by these KIH-based molecules on NPs. The radii of the KIH-based pMHCs on NPs is larger than those of their zippered counterparts. This may decrease the occurrence of potentially disruptive interactions of the TCR-binding N-terminal pMHC domain with the NP during the functionalization process. In this regard, the KIH portion of the KIH-based pMHC molecule might buffer conformational changes on the pMHC’s N-terminal domain imposed by the NP. In addition, the disulfide bonds that are introduced into these KIHs might contribute to the hypothetical resistance of these molecules to allosteric changes induced by neighboring pMHC molecules or the NP itself. Whatever the precise explanation, at a practical level, these molecular modifications actually change the minimal optimal pMHC density threshold for pharmacodynamic activity in vivo; the “32” minimal optimal pMHC valency threshold defined for 20-nm diameter NPs displaying conventional pMHCs appears to be lower for Cys-trapped, KIH-based pMHCs^4^.

### hIgG1 KIH-based stabilization of peptide-HLA-DQ molecules

Peptide-HLA-DQ complexes are difficult to express^22^. As noted above, we could only produce significant amounts of c-jun/c-fos-zippered Gliadin_62–72_/DQB1*0201/DQA1*0501 when the peptide was cys-trapped onto the MHC molecule, albeit at low yields (Table 1 and Fig. 5b). Remarkably, substitution of the leucine zipper domain for a KIH enabled the production of Gliadin_62–72_/DQB1*0201/DQA1*0501 by CHO-S cells at yields that were 15-fold higher (Table 1 and Fig. 9f).

### KIH-based stabilization of empty MHC class II molecules

Some experimental approaches for T-cell epitope mapping require the use of extensive arrays of pMHCII tetramers to identify epitope reactivity by flow cytometry^23–25^. In this context, the use of pMHCII molecules displaying covalently tethered peptides is not practical, as it implies purifying many different pMHCII molecules and generating the corresponding fluorochrome-labeled tetramers for each specific epitope. We thus investigated if the KIH-based approach could also be used to express high levels of non-peptide-tethered pMHCIIs from CHO cells and whether these compounds could be used for peptide-loading in vitro^26,27^. As shown in Table 1, transduced CHO-S cells secreted high levels of four different non-peptide-tethered human DRB types, including DRB1*0301/DRA1*0101, DRB4*0101/DRA*0101, DRB5*0101/DRA*0101, and DRB1*1501/DRA*0101. Importantly, these complexes could be loaded with peptides in vitro and the corresponding tetramers bound to cognate T-cells essentially like their peptide-tethered counterparts (Fig. 9g, h).

### Amplification of KIH-based pMHCII multimer staining

Different strategies have been described to increase the staining intensity cognate T-cells with fluorochrome-labeled pMHC multimers^28^, including the use of kinase inhibitors, the formation of cooperative pMHC/TCR clusters with crosslinking antibodies^29^, and the use of scaffolds enabling the production of higher-order multimeric structures such as dextramers^30^. This is particularly useful in autoimmune diseases, where the peripheral frequencies of autoreactive T-cells and their avidity for cognate pMHC complexes are significantly lower than those seen for foreign antigen-specific T-cells, such as in the context of infection and allergy. We thus investigated whether the signal-to-noise ratio of cognate T-cell staining with pMHCII tetramers could be improved using anti-hIgG-based amplification of KIH-based pMHCII tetramer binding. Human PBMCs were spiked with human clonal IGRP_13–25_/DR3-specific CD4^+^ T-cells and stained with cognate KIH-based pMHCII tetramers in the presence or absence of the protein kinase inhibitor Dasatinib (to inhibit TCR downregulation) followed by anti-hIgG-PE amplification. As shown in Fig. 9i, anti-hIgG increased the mean fluorescence signal intensity of tetramer staining, both in the presence and absence of Dasatinib.

## Discussion

We have described a novel pMHCII heterodimerization strategy that enables the production and purification, at high yields, of stable pMHCII monomers for a variety of applications. In this novel approach, we replace the transmembrane and cytoplasmic regions of the MHCII α and β chains for the human or mouse IgG1-Fc modified to form knobs and holes, respectively. These molecules are invariably SDS stable, express at significantly higher levels than conventional leucine-zippered pMHCIIs and can be easily purified from culture supernatants using protein A/G chromatography without the need to include foreign, immunogenic affinity-purification tags in the molecule. In addition, they have superior TCR binding and triggering properties and, when used as multimeric structures to enumerate antigen-specific T-cells in complex biological samples, are amenable to signal amplification, including the use of anti-hFc antibodies. Collectively, the advantages of the molecular pMHCII engineering approach described herein overcome the roadblocks that currently preclude the use of pMHCII designs for therapeutic applications. In addition, the high expression yields of non-peptide-tethered KIH-based pMHCs should facilitate the screening of epitope libraries in the context of specific MHCII molecules and antigen receptors. This KIH approach could also help stabilize difficult-to-express soluble TCRαβ heterodimers for multiple uses, including the identification of specific pMHC targets^31^.

## Methods

### Mice

NOD/Lt and BDC2.5-NOD mice (expressing a transgenic T-cell receptor for the BDC2.5mi/IA^g7^ complex)^32^ were purchased from the Jackson Lab (Bar Harbor, ME). Mice were housed and bred in a specific pathogen-free facility at the Cumming School of Medicine at the University of Calgary. The animal experiments described herein were approved by the University of Calgary Animal Care Committee and complied with the Canadian Council of Animal Care.

### pMHC production

Recombinant pMHCII were produced in CHO-S cells (Invitrogen) transduced with lentiviruses (Vector Builder, Chicago, IL) encoding a monocistronic message in which the peptide-MHCβ (or non-peptide-tethered MHCβ) and MHCα chains were separated by a ribosome skipping P2A-coding sequence, followed by an IRES-EGFP cassette^33^. Alternatively, the peptide-MHCβ and MHCα chains were encoded in separate lentiviruses encoding IRES-EGFP and IRES-CFP cassettes, respectively. Peptide-tethered MHCII molecules were biotinylated in vitro, as described below. “Empty” MHCII molecules were biotinylated in vivo, by expressing the corresponding lentivirus-transduced constructs in BirA-transgenic CHO cells (see below).

To express the various pMHCIIs, transduced CHO-S cells were grown in 2 L baffled flasks (Nalgene) in a shaker incubator at 125 rpm, 5% CO_2_ and 37 °C. Basal medium was Power-CHO-2 (Lonza) supplemented with 8 mM glutamine (Lonza) and gentamicin sulfate (0.05 mg/mL) (Lonza). The cultures were started in 400 mL of basal medium at 350,000–400,000 cells/mL and were supplemented with feeds: Cell Boost 7a (Hyclone) at 3% v/v and Cell Boost 7b (Hyclone) at 0.3% v/v on days 0, 3, 4, 5, 6, 8, 9, and 10. A temperature shift to 34 °C was done when cell densities reached 5–7 × 10^6^ cells/mL. Additional glutamine was added on day 7, to 2 mM. Glucose was added to 4.5 g/L when levels dropped below 3.5 g/L. Cells were harvested on Day 14 or when cell viability fell below 60%.

The secreted proteins were purified by sequential affinity chromatography on nickel and strep-tactin columns (for c-fos/c-jun-based pMHCII), protein A/G columns (for KIH-based pMHCII), and avidin columns (for in vivo-biotinylated empty KIH-based pMHCIIs after protein A/G purification) and used for NP coating or biotinylated in vitro (for peptide-tethered pMHCII) to produce pMHC tetramers using fluorochrome-conjugated streptavidin.

### Molecular modeling

Molecular modeling was done with the DeepView-Swiss-PdbViewer software^34^. KIH heterodimer modeling was based on the published crystal structure^35^ (Protein Data Bank (PDB) ID: 4NQS). The pMHCII cys-trap model was based on the published crystal structure of IA^g7^ complexed with GAD_207–220_ (PDB ID: 1ES0)^36^.

### SDS-PAGE

The proteins were electrophoresed in 12% SDS-PAGE gels. To evaluate SDS stability of pMHCII monomers, samples were loaded with 0.83% SDS and were either boiled (100 °C for 5 min) or left unboiled. Fully denaturing conditions involved the addition of 20 mM β2-ME (Sigma). All uncropped PAGE gel images are shown in Supplementary Fig. 1.

### In vitro biotinylation of pMHCII monomers

Biotinylation of pMHCII was done by using a biotin-protein ligase kit (BirA enzyme, Avidity). Briefly, 25 μM of pMHC was biotinylated with 10 μg of BirA enzyme in 50 mM bicine buffer pH 8.3 with 10 mM magnesium acetate, 10 mM ATP, and 85 μM of d-biotin at room temperature overnight. The reaction mixture was dialyzed against 20 mM Tris-HCl buffer pH 8 and the resulting pMHCII was purified by ion exchange (mono-Q) chromatography. Biotin-conjugated pMHCII fractions were identified via ELISA using horseradish peroxidase-streptavidin (Sigma) and characterized via denaturing SDS-PAGE. The biotin-conjugated pMHCII fractions were pooled, buffer exchanged into PBS by spin ultrafiltration (Millipore, MW cut-off 30 KDa) and stored at −80 °C.

### pMHC tetramers

Phycoerythrin (PE)-conjugated tetramers were prepared using biotinylated pMHCII monomers and used to stain peripheral T-cells or TCR-transfected Jurkat cell lines^37,38^. “Empty” MHCII complexes were biotinylated in BirA-transgenic CHO-S cells and purified on avidin columns. Briefly, CHO-S cells were transduced with lentiviruses encoding each of the two chains of the KIH constructs as described above. BirA-ER enzyme (Addgene) was cloned into another lentiviral plasmid carrying human CD4 as a reporter gene and used to transduce CHO-S cell lines expressing the different KIH-based MHCIIs. Cells were FACS-sorted based on positivity for GFP, CFP, and human CD4 using a Becton Dickinson FACsAriaII sorter. Cell lines were expanded and grown to a density of 10–15^7^ cells/mL during 14 days in the presence of 2 µg/mL of biotin (ThermoFisher). Biotinylated soluble MHCII molecules were purified from culture supernatants by protein G column chromatography using an ÄKTA protein purification system (GE). PBS or 20 mM Trizma buffer exchange was done using a size exclusion column (GE). A second purification using avidin column kit was done in order to purify in vivo-biotinylated proteins (ThermoFisher).

The biotinylated molecules were then loaded with peptide by incubation with a 10-fold molar excess of PDC-E2_122–135_ (Tebu-bio), PDC-E2_249–262_ (Tebu-bio), and IGRP_13–25_ (Genscript) in 100 mM NaPO_4_ pH 6.0, 0.2% n-octyl-d-glucopyranoside (Sigma-Aldrich) and 1 mg/ml Pefabloc® (Sigma-Aldrich) for 72 h at 37 °C. The peptide-loaded MHCII molecules were then incubated with PE-streptavidin (ThermoFisher) at a 5:1 molar ratio overnight at room temperature to generate tetrameric pMHCII complexes.

### Flow cytometry

To stain mononuclear cell suspensions from NOD mice, peripheral blood, splenocytes, lymph node and bone marrow cell suspensions were incubated with avidin for 15 min at room temperature and stained with tetramer (10–33 µg/mL, see below) in FACS buffer (0.05% sodium azide and 1% FBS in PBS) for 30 min at 4 °C, washed, and incubated with FITC-conjugated anti-CD4 (5 µg/mL) and PerCP-conjugated anti-B220 (2 µg/mL; as a ‘dump’ channel) for 30 min at 4 °C, in the presence of an anti-CD16/CD32 mAb (2.4G2; BD Pharmingen) to block FcRs. Cells were washed, fixed in 1% paraformaldehyde (PFA) in PBS and analyzed with FACScan, FACSaria, BD LSRII, FACSCanto or Fortessa flow cytometers. Analysis was done using FlowJo software.

TCR-transduced Jurkat cell lines were stained with 10 μg/ml of c-jun/c-fos-based pMHCII tetramer or 33 μg/ml KIH-based tetramer in 50 μl of PBS for 1 h at 37 °C. Propidium iodide (Sigma, St. Louis, Missouri, USA) was added 5 min before analysis to discriminate live from dead cells.

For experiments using PBMCs spiked with clonal cells, experimental samples were created by mixing clonal T-cells (10^4^) with PBMCs (10^6^). The PBMCs were HLA-matched for the restricting HLA of the T-cell clone used. Some samples were treated prior to tetramer staining with the protein kinase inhibitor (PKI) Dasatinib (Axon Medchem) at 50 nM for 30 min at 37 °C. Tetramer staining was performed with 33 μg/ml of peptide-loaded KIH-based pMHC tetramers in 50 μl of PBS for 1 h at 37 °C. All samples were subsequently stained with anti-CD4 (αCD4) (OKT4; BioLegend), to gate on CD4^hi^ cells and, in some cases, with anti-human Fc-PE (Jackson ImmunoResearch) for 20 min at 37 °C. Propidium iodide (Sigma) was added 5 min before analysis to discriminate live from dead cells. FACS gating strategies are provided in Supplementary Fig. 2.

### NP synthesis

Maleimide-functionalized, pegylated iron oxide NPs (PFM series) were produced in a single-step thermal decomposition in the absence of surfactants^4^. Briefly, 3 g Maleimide-PEG (2 kDa MW, Jenkem Tech, USA) were melted in a 50 mL round bottom flask at 100 °C and then mixed with 7 mL of benzyl ether and 2 mmol Fe(acac)_3_. The reaction was stirred for 1 h and heated to 260 °C with reflux for 2 h. The mixture was cooled to room temperature and mixed with 30 mL water. Insoluble materials were removed by centrifugation at 2000 × *g* for 30 min. The NPs were purified using magnetic (MACS) columns (Miltenyi Biotec) and stored in water at room temperature or 4 °C. The concentration of iron was determined spectrophotometrically at 410 nm in 2 N hydrochloric acid (HCl).

### pMHCII conjugation to NPs

pMHCII conjugation to maleimide-functionalized NPs (PF-M) was done via the free C-terminal Cys engineered into the MHCα chain/Knob. Briefly, pMHCs were mixed with NPs in 40 mM phosphate buffer, pH 6.0, containing 2 mM ethylenediaminetetraacetic acid (EDTA), 150 mM NaCl, and incubated overnight at room temperature. pMHC-conjugated NPs were purified by magnetic separation and concentrated by ultrafiltration through Amicon Ultra-15 (100–300 kDa cut-off) and stored in PBS.

### NP characterization

The size and dispersity of unconjugated and pMHCII-conjugated NPs were assessed via transmission electron microscopy (TEM, Hitachi H7650) and dynamic light scattering (DLS, Zetasizer, Malvern). Pegylated and pMHC-NPs were analyzed via 0.8% agarose gel electrophoresis, native and denaturing 10% SDS-PAGE. To quantify pMHC valency, we measured the pMHC concentration of the pMHC-NP preps using the Bradford assay (Thermo Scientific).

### Reactivity of hMHCIIs to conformation epitope-specific mAbs

The KIH-based pMHC monomers were diluted to an identical concentration (200 ng/mL) and serially diluted. A sandwich ELISA assay was used to capture and quantify the pMHCs. Briefly, plates were coated with goat anti-human IgG (Jackson ImmunoResearch) (working concentration 24 μg/mL) as a capture antibody. The capture antibody (100 μL/well) was incubated in a 96-well flat bottom Immuno plate (Thermo Scientific) overnight at room temperature. The plates were blocked using PBS containing 1% BSA and 0.05% sodium azide for 1 h. The plates were then washed four times with PBS containing 0.5% Triton X-100, 200 μL/well (washing buffer). The serially diluted pMHC-human KIH fusion protein solution (100 μL/well) was added to the wells and incubated for 2 h at room temperature. The plates were washed four times. The captured pMHCIIs were then detected using biotinylated anti-human HLA-DR mAb (clone L243, from Biolegend; 0.4 μg/well, 100 μL/well). The plates were incubated with the capture antibody for 2 h at room temperature, washed four times and then incubated with ExtrAvidin Peroxidase Conjugate (Sigma-Aldrich; 1:2000 dilution in PBS, 100 μL/well) for 30 min at room temperature. The plates were washed again, and incubated with 3,3′,5,5′-Tetramethylbenzidine (TMB, Sigma-Aldrich; 100 μL/well) for 5 min. The color reaction was stopped by adding 50 μL of 2 N H_2_SO_4_. The absorbance of the reaction was measured at 450 nm and 570 nm wavelengths using a plate reader (SpectraMax i3x, Molecular Devices).

### TCR signaling in TCR/CD4-transfected Jurkat cells

The TCRα and TCRβ cDNAs encoding the BDC2.5-TCR were generated from BDC2.5-CD4^+^ T-cell-derived mRNA using the 5′ RACE System for Rapid Amplification of cDNA Ends, version 2.0 kit (Thermo-Fisher Scientific), and subcloned as a P2A-tethered single open-reading frame into a retroviral vector upstream of an IRES-eGFP cassette. The TCR cDNAs encoding human IGRP_13–25_/DR3-, PDC-E2_122–135_/DRB4*0101/DRA1*-0101-, and PDC-E2_249–262_/DRB4*0101/DRA1*-0101-specific TCRs were cloned from human T-cell clones generated from T1D or primary biliary cholangitis (PBC) patients^4^. Briefly, PBMCs, obtained from PBC patients recruited under informed consent approved by the Institutional Review Board at Hospital Clinic, were isolated from heparinized blood by gradient centrifugation and resuspended at 5 × 10^6^/mL in RPMI-1640 media supplemented with 10% human AB serum. The cells were cultured in 24-well plates in the presence of 10 μg/mL peptide. After 7–10 days, cells were washed, and cultured for 5 days in wells coated, at high density, with avidin and biotinylated pMHCII monomer. Finally, cells were cultured for 5 additional days in the presence of 1 μg/mL of soluble anti-hCD28 mAb (BD Pharmingen) followed by interleukin-2 (R&D). Tetramer^+^ CD4^+^ T-cells were single-cell sorted into 96-well plates using a FACSAriaII sorter (Becton Dickinson) and used for TCR sequencing^4^.

The human CD3^+^/TCRβ^−^ JurMA (Jurkat) reporter cell line (expressing NFAT-driven luciferase) was transduced with retroviruses encoding mouse or human CD4 and mouse or human TCRαβ, respectively. eGFP and mouse or human CD4 double-positive cells were sorted by flow cytometry and stained with PE-labeled pMHCII tetramers to confirm specificity.

To measure NFAT-driven expression of luciferase, wild-type and BDC2.5/mCD4^+^ or IGRP_13–35_/DR3-TCR/hCD4^+^ Jurkat cells were plated at 500,000 cells/mL in 200 µl of DMEM (Sigma-Aldrich) supplemented with 10% FBS (Sigma-Aldrich) in the presence or absence of 10 μg/mL of anti-hCD3ε mAb (OKT3, BD Biosciences) or various concentrations of pMHC-coated PFM for 12 h. Cells were washed three times with PBS and 10^5^ cells lysed in 20 µl Cell Culture Lysis Reagent (Promega) and incubated with 100 µl of Luciferase Assay Reagent (Promega) in opaque white plates (Greiner Bio One International GmbH) using a Veritas™ Microplate Luminometer (Promega) with injectors. Luciferase activity was expressed as relative luminescence units (RLUs), normalized to the luciferase activity of anti-CD3ε mAb-challenged cells.

### pMHCII-NP therapy of NOD mice

Cohorts of 10-week-old female NOD mice were injected i.v. with pMHCII-coated NPs in PBS (20 μg pMHC/dose) twice a week for 5 weeks. Increases in the size of tetramer^+^ CD4^+^ T-cell pools in blood, spleen, lymph nodes and/or marrow, as well as their phenotypic properties, were assessed by flow cytometry as described^2^.

### Cytokine secretion assay

CD4^+^ T-cells from pMHC-NP-treated mice were enriched from spleen cell suspensions using a BD Imag enrichment kit, stained with pMHCII tetramers as described above and sorted into tetramer^+^ and tetramer^−^ subsets by flow cytometry. FACS-sorted cells (2–3 × 10^4^) were stimulated with anti-CD3/anti-CD28 mAb-coated beads for 48 h and the supernatants collected 48 h later for measurement of cytokines via Luminex.

### Statistical analyses

Quantitative data were compared by Mann–Whitney U. Statistical significance was assumed at *P* < 0.05.

### Reporting summary

Further information on research design is available in the Nature Research Reporting Summary linked to this article.

## Supplementary information

Supplementary Figures 1 and 2

Reporting Summary

source data

## Data Availability

The source data underlying Figs. 7b–e, 8a–d, 9c–e and 10a–d are provided as a Source Data File. Uncropped gel images and FACS gating strategies are provided in the Supplementary Information.

## References

[1] Altman JD (1996). Phenotypic analysis of antigen-specific T lymphocytes. Science.

[2] Clemente-Casares X (2016). Expanding antigen-specific regulatory networks to treat autoimmunity. Nature.

[3] Tsai S (2010). Reversal of autoimmunity by boosting memory-like autoregulatory T cells. Immunity.

[4] Singha S (2017). Peptide-MHC-based nanomedicines for autoimmunity function as T-cell receptor microclustering devices. Nat. Nanotechnol.

[5] Umeshappa CS (2019). Supression of a broad spectrum of liver autoimmune pathologies by single peptide-MHC-based nanomedicines. Nat. Commun..

[6] Cosson P, Bonifacino JS (1992). Role of transmembrane domain interactions in the assembly of class II MHC molecules. Science.

[7] Vollers SS, Stern LJ (2008). Class II major histocompatibility complex tetramer staining: progress, problems, and prospects. Immunology.

[8] Merchant AM (1998). An efficient route to human bispecific IgG. Nat. Biotechnol..

[9] Carter P (2001). Bispecific human IgG by design. J. Immunol. Methods.

[10] Sadegh-Nasseri S, Germain RN (1991). A role for peptide in determining MHC class II structure. Nature.

[11] Sadegh-Nasseri S, Germain RN (1992). How MHC class II molecules work: peptide-dependent completion of protein folding. Immunol. Today.

[12] Verreck FA (1996). The generation of SDS-stable HLA DR dimers is independent of efficient peptide binding. Int. Immunol..

[13] Nelson CA, Petzold SJ, Unanue ER (1993). Identification of two distinct properties of class II major histocompatibility complex-associated peptides. Proc. Natl Acad. Sci. USA.

[14] Stadinski BD (2010). Diabetogenic T cells recognize insulin bound to IAg7 in an unexpected, weakly binding register. Proc. Natl Acad. Sci. USA.

[15] Yang J (2014). Autoreactive T cells specific for insulin B:11-23 recognize a low-affinity peptide register in human subjects with autoimmune diabetes. Proc. Natl Acad. Sci. USA.

[16] Casares S (2002). Down-regulation of diabetogenic CD4+ T cells by a soluble dimeric peptide-MHC class II chimera. Nat. Immunol..

[17] Preda I (2005). Soluble, dimeric HLA DR4-peptide chimeras: an approach for detection and immunoregulation of human type-1 diabetes. Eur. J. Immunol..

[18] Moro M (2005). Generation of functional HLA-DR*1101 tetramers receptive for loading with pathogen- or tumour-derived synthetic peptides. BMC Immunol..

[19] Goldberg B, Bona C (2011). Dimeric MHC-peptides inserted into an immunoglobulin scaffold as new immunotherapeutic agents. J. Cell. Mol. Med..

[20] Gorga JC, Horejsi V, Johnson DR, Raghupathy R, Strominger JL (1987). Purification and characterization of class II histocompatibility antigens from a homozygous human B cell line. J. Biol. Chem..

[21] Stockel J (1994). Refolding of human class II major histocompatibility complex molecules isolated from *Escherichia coli*. Assembly of peptide-free heterodimers and increased refolding-yield in the presence of antigenic peptide. J. Biol. Chem..

[22] Quarsten H (2001). Staining of celiac disease-relevant T cells by peptide-DQ2 multimers. J. Immunol..

[23] James EA (2007). Tetramer-guided epitope mapping reveals broad, individualized repertoires of tetanus toxin-specific CD4+ T cells and suggests HLA-based differences in epitope recognition. Int. Immunol..

[24] Novak EJ (2001). Tetramer-guided epitope mapping: rapid identification and characterization of immunodominant CD4+ T cell epitopes from complex antigens. J. Immunol..

[25] Novak EJ, Liu AW, Nepom GT, Kwok WW (1999). MHC class II tetramers identify peptide-specific human CD4(+) T cells proliferating in response to influenza A antigen. J. Clin. Invest..

[26] Wallny HJ, Sollami G, Karjalainen K (1995). Soluble mouse major histocompatibility complex class II molecules produced in *Drosophila* cells. Eur. J. Immunol..

[27] Yang J, Jaramillo A, Shi R, Kwok WW, Mohanakumar T (2004). In vivo biotinylation of the major histocompatibility complex (MHC) class II/peptide complex by coexpression of BirA enzyme for the generation of MHC class II/tetramers. Hum. Immunol..

[28] Dolton G (2015). More tricks with tetramers: a practical guide to staining T cells with peptide-MHC multimers. Immunology.

[29] Lissina A (2009). Protein kinase inhibitors substantially improve the physical detection of T-cells with peptide-MHC tetramers. J. Immunol. Methods.

[30] Batard P (2006). Dextramers: new generation of fluorescent MHC class I/peptide multimers for visualization of antigen-specific CD8+ T cells. J. Immunol. Methods.

[31] Crawford F (2006). Use of baculovirus MHC/peptide display libraries to characterize T-cell receptor ligands. Immunol. Rev..

[32] Katz JD, Wang B, Haskins K, Benoist C, Mathis D (1993). Following a diabetogenic T cell from genesis through pathogenesis. Cell.

[33] Holst J (2006). Generation of T-cell receptor retrogenic mice. Nat. Protoc..

[34] Guex N, Peitsch MC (1997). SWISS-MODEL and the Swiss-PdbViewer: an environment for comparative protein modeling. Electrophoresis.

[35] Elliott JM (2014). Antiparallel conformation of knob and hole aglycosylated half-antibody homodimers is mediated by a CH2-CH3 hydrophobic interaction. J. Mol. Biol..

[36] Corper AL (2000). A structural framework for deciphering the link between I-Ag7 and autoimmune diabetes. Science.

[37] Amrani A (2000). Progression of autoimmune diabetes driven by avidity maturation of a T-cell population. Nature.

[38] Stratmann T (2000). The I-Ag7 MHC class II molecule linked to murine diabetes is a promiscuous peptide binder. J. Immunol..

