# Supplementary material for: Increased yields and biological potency of knob-into-hole-based soluble MHC class II molecules
Source: Nat Commun. 2019 Oct 29;10:4917. doi: 10.1038/s41467-019-12902-2 (PMC6820532; doi:10.1038/s41467-019-12902-2)
Supplement: Supplementary file 1 — Supplementary Figures 1 and 2 [file 41467_2019_12902_MOESM1_ESM.pdf]

SUPPLEMENTARY INFORMATION

INCREASED STABILITY, YIELDS AND BIOLOGICAL POTENCY OF KNOB-INTO-HOLE-BASED SOLUBLE MAJOR HISTOCOMPATIBILITY COMPLEX CLASS II MOLECULES

Serra et al.

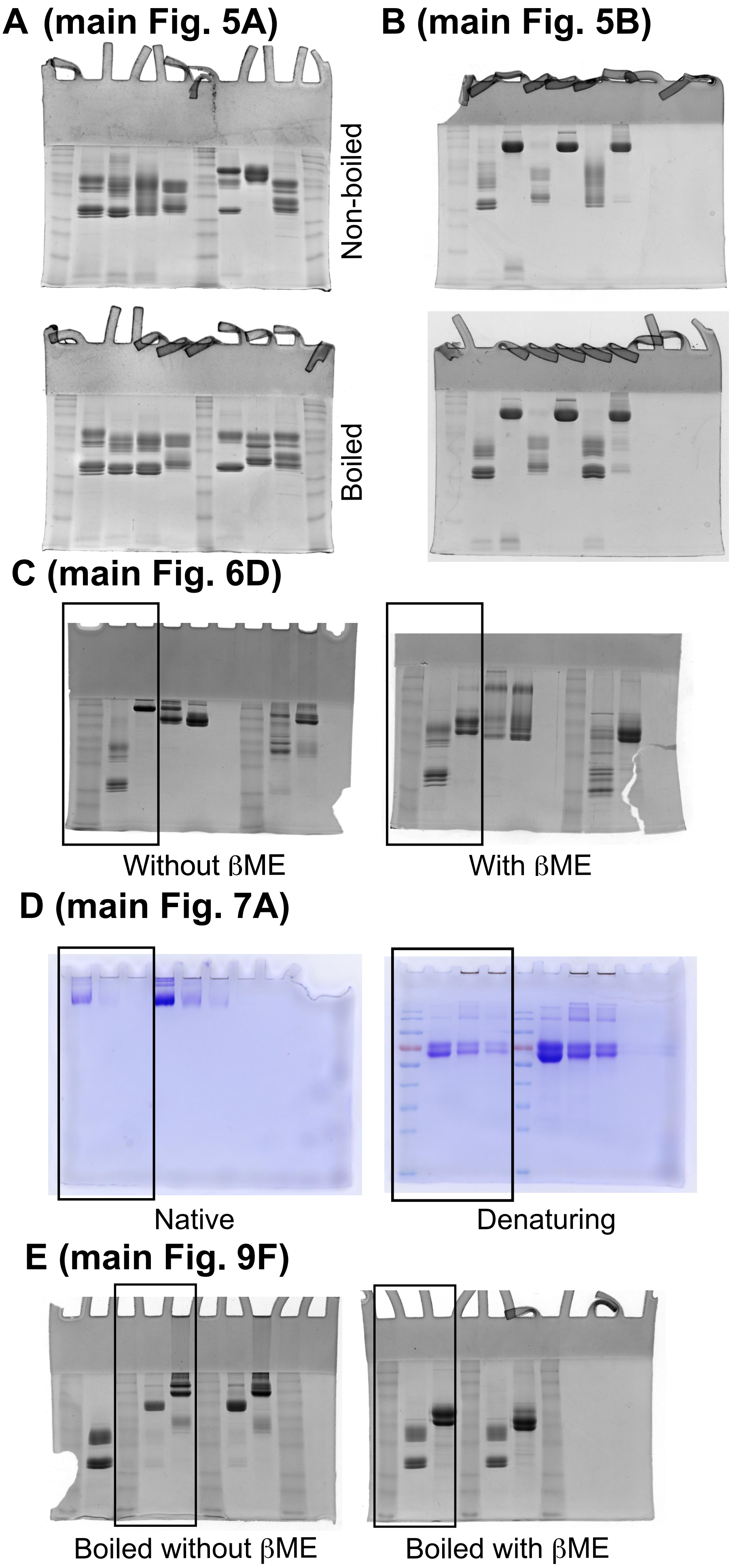

Supplementary Figure 1. Uncropped SDS-PAGE gels corresponding to the stated Figure panels.

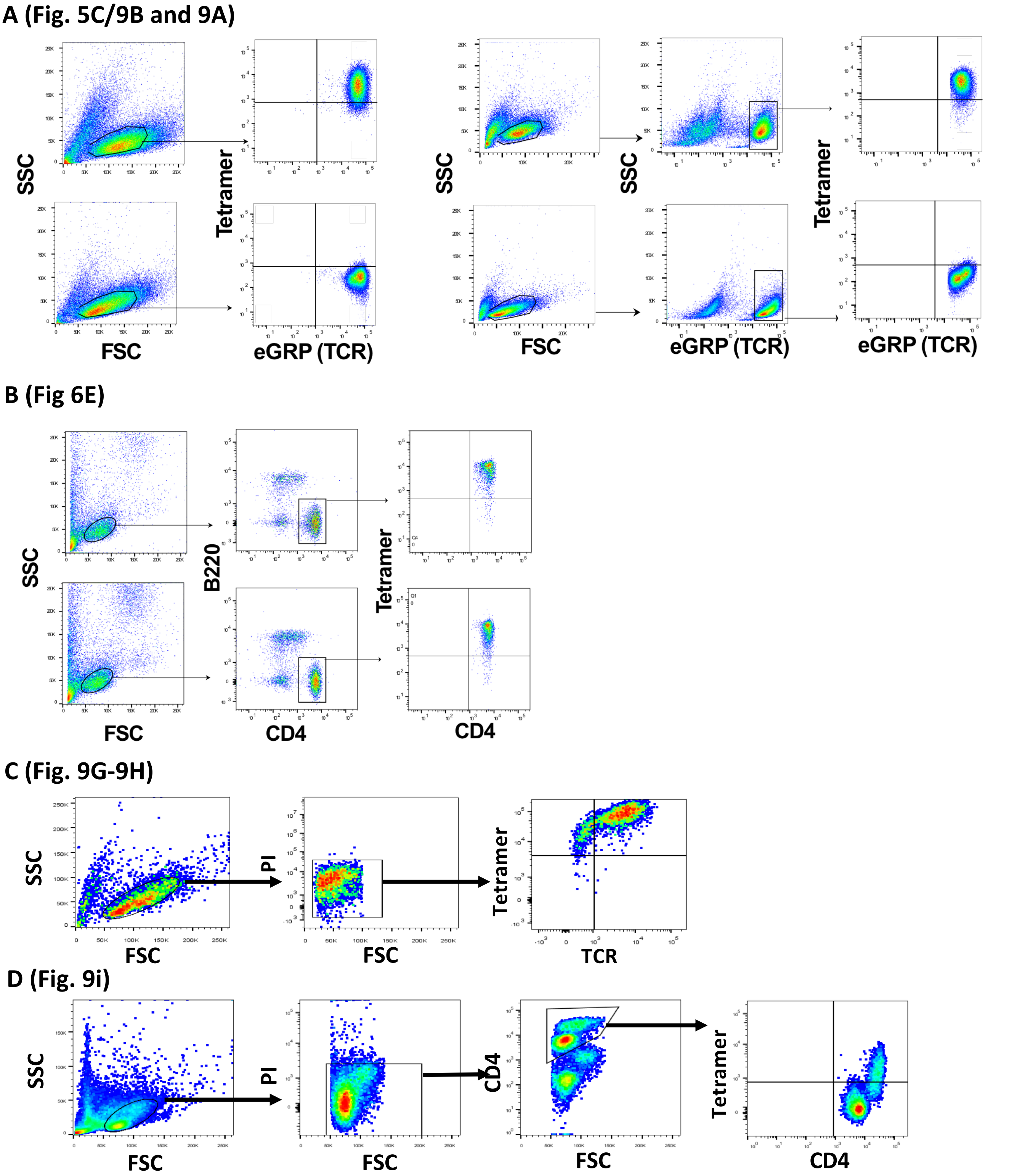

Supplementary Figure 2. Gating strategies for FACS analysis. A. Gating strategy to analyze the TCR-transduced Jurkat cell lines shown on Figs. 5C and 9B (left), and 9A (right). **B**, Gating strategy to analyze the CD4+ T-cells of BDC2.5 TCR-transgenic NOD mice. **C.** Gating strategy to analyze the TCR-transduced Jurkat cell lines shown on Figs. 9G and 9H. **D.** Gating strategy to analyze human peripheral blood mononuclear cells (PBMCs) spiked with clonal CD4+ T-cells shown on Fig. 9**I**.
