# Supplementary material for: Increased yields and biological potency of knob-into-hole-based soluble MHC class II molecules
Source: Nat Commun. 2019 Oct 29;10:4917. doi: 10.1038/s41467-019-12902-2 (PMC6820532; doi:10.1038/s41467-019-12902-2)
Supplement: Supplementary file 2 — Reporting Summary [file 41467_2019_12902_MOESM2_ESM.pdf]

nature research

Corresponding author(s): Pere Santamaria and Pau Serra

Last updated by author(s): October 1,2019

Reporting Summary

Nature Research wishes to improve the reproducibility of the work that we publish. This form provides structure for consistency and transparency in reporting. For further information on Nature Research policies, see Authors & Referees and the Editorial Policy Checklist.

Statistics

For all statistical analyses, confirm that the following items are present in the figure legend, table legend, main text, or Methods section.

n/a	Confirmed	
☐	☒	The exact sample size (*n*) for each experimental group/condition, given as a discrete number and unit of measurement
☐	☒	A statement on whether measurements were taken from distinct samples or whether the same sample was measured repeatedly
☐	☒	The statistical test(s) used AND whether they are one- or two-sided<br>*Only common tests should be described solely by name; describe more complex techniques in the Methods section.*
☒	☐	A description of all covariates tested
☒	☐	A description of any assumptions or corrections, such as tests of normality and adjustment for multiple comparisons
☐	☒	A full description of the statistical parameters including central tendency (e.g. means) or other basic estimates (e.g. regression coefficient) AND variation (e.g. standard deviation) or associated estimates of uncertainty (e.g. confidence intervals)
☐	☒	For null hypothesis testing, the test statistic (e.g. *F*, *t*, *r*) with confidence intervals, effect sizes, degrees of freedom and *P* value noted<br>*Give P values as exact values whenever suitable.*
☒	☐	For Bayesian analysis, information on the choice of priors and Markov chain Monte Carlo settings
☒	☐	For hierarchical and complex designs, identification of the appropriate level for tests and full reporting of outcomes
☒	☐	Estimates of effect sizes (e.g. Cohen's *d*, Pearson's *r*), indicating how they were calculated

Our web collection on statistics for biologists contains articles on many of the points above.

Software and code

Policy information about availability of computer code

Data collection	FlowJo, Prism 8
Data analysis	Prism 8

For manuscripts utilizing custom algorithms or software that are central to the research but not yet described in published literature, software must be made available to editors/reviewers. We strongly encourage code deposition in a community repository (e.g. GitHub). See the Nature Research guidelines for submitting code & software for further information.

Data

Policy information about availability of data

All manuscripts must include a data availability statement. This statement should provide the following information, where applicable:

- Accession codes, unique identifiers, or web links for publicly available datasets
- A list of figures that have associated raw data
- A description of any restrictions on data availability

Raw data used to build the figures are available upon request

Field-specific reporting

Please select the one below that is the best fit for your research. If you are not sure, read the appropriate sections before making your selection.

☒ Life sciences ☐ Behavioural & social sciences ☐ Ecological, evolutionary & environmental sciences

For a reference copy of the document with all sections, see nature.com/documents/nr-reporting-summary-flat.pdf

Life sciences study design

All studies must disclose on these points even when the disclosure is negative.

Sample size	Based on previous studies in similar biological systems. Sample sizes were as large as possible with tight controls of gender, age, dosing regiments, and experimental conditions within and between experiments, to limit experimental variability. In vitro experiments typically involved smaller sample sizes than in vivo experiments and all statistically significant differences are reported with the corresponding P values.
Data exclusions	No data/mice were excluded from analyses. All analyzed samples are reported.
Replication	The conclusions of independent experiments using specific pMHCIIs were validated using several other pMHCII types.
Randomization	Not applicable
Blinding	The investigators were not blinded during data collection and analysis, except for cytokine measurements.

Reporting for specific materials, systems and methods

We require information from authors about some types of materials, experimental systems and methods used in many studies. Here, indicate whether each material, system or method listed is relevant to your study. If you are not sure if a list item applies to your research, read the appropriate section before selecting a response.

Materials & experimental systems

n/a	Involved in the study	
☐	☒	Antibodies
☐	☒	Eukaryotic cell lines
☒	☐	Palaeontology
☐	☒	Animals and other organisms
☐	☒	Human research participants
☒	☐	Clinical data

Methods

n/a	Involved in the study	
☒	☐	ChIP-seq
☐	☒	Flow cytometry
☒	☐	MRI-based neuroimaging

Antibodies

Antibodies used	All antibodies were obtained form commercial sources and the clone numbers and source identified in the Methods..
Validation	Validation was from the commercial supplier and/or the published scientific literature using the exact same antibodies.

Eukaryotic cell lines

Policy information about cell lines

Cell line source(s)	Source of all cell lines and cell line identity are indicated within the manuscript.
Authentication	CHO cells were commercially available and were not authenticated in our laboratories upon receipt. They were used for pMHC production. However, we regularly sequence transduced pMHC-production cell lines (the transduced genes), to confirm identity in terms of pMHC specificity.
Mycoplasma contamination	Cell lines used for pMHC production were free of mycoplasma contamination.
Commonly misidentified lines (See ICLAC register)	Not Applicable.

Animals and other organisms

Policy information about studies involving animals; ARRIVE guidelines recommended for reporting animal research

Laboratory animals	Mouse gender and age are available in the figure legends and/or methods. All studies were approved by the corresponding institutional animal care committees.
Wild animals	Not applicable.
Field-collected samples	Not applicable.

Ethics oversight	Animal Care Committee of the Cumming School of Medicine at the University of Calgary.

Note that full information on the approval of the study protocol must also be provided in the manuscript.

Human research participants

Policy information about studies involving human research participants

Population characteristics	The patients from whom we derived T-cell clones for this study signed an informed consent form and were informed of the objectives of the study before samples were collected. The consent form was approved by the IDIBAPS Institutional Review Board.
Recruitment	Patients were recruited into providing samples for HLA typing by the attending physician during a regular visit. Only patients carrying the HLA alleles of interest were recruited.
Ethics oversight	IDIBAPS Institutional Review Board at Hospital Clinic de Barcelona.

Note that full information on the approval of the study protocol must also be provided in the manuscript.

Flow Cytometry

Plots

Confirm that:

- [x] The axis labels state the marker and fluorochrome used (e.g. CD4-FITC).
- [x] The axis scales are clearly visible. Include numbers along axes only for bottom left plot of group (a 'group' is an analysis of identical markers).
- [x] All plots are contour plots with outliers or pseudocolor plots.
- [x] A numerical value for number of cells or percentage (with statistics) is provided.

Methodology

Sample preparation	This is described in the Methods section.
Instrument	The instruments used are indicated in the Methods section.
Software	FlowJo
Cell population abundance	Not applicable
Gating strategy	The various gating strategies are provided in Supplementary Figure 2.

- [x] Tick this box to confirm that a figure exemplifying the gating strategy is provided in the Supplementary Information.
